# 1022. The Role of Baseline Body Temperature in Neutropenic Fever

**DOI:** 10.1093/ofid/ofac492.863

**Published:** 2022-12-15

**Authors:** Anthony J Corsi, Madison Searles, Christina Lupone, Ivayla I Geneva

**Affiliations:** State University of New York - Upstate, Syracuse, New York; State University of New York - Upstate, Syracuse, New York; State University of New York - Upstate, Syracuse, New York; Crouse Hospital, SYRACUSE, New York

## Abstract

**Background:**

The management of neutropenic fever patients is challenging – from identification to diagnosis to treatment. We hypothesize that patients’ individual baseline body temperature provides diagnostic and prognostic value.

**Methods:**

This is an analysis of 92 adult patients admitted for neutropenic fever to a tertiary medical center over 1 year period. We modelled the length of stay and the ability to find a definitive diagnosis using the change in body temperature from each patient’s outpatient baseline, the neutropenia level and overall patient acuity on admission, persistence of fever over 48-72 hours, and age. All temperatures were standardized to oral. Refer to Table 1 for the inclusion and exclusion criteria and statistical methods.

**Table 1.**
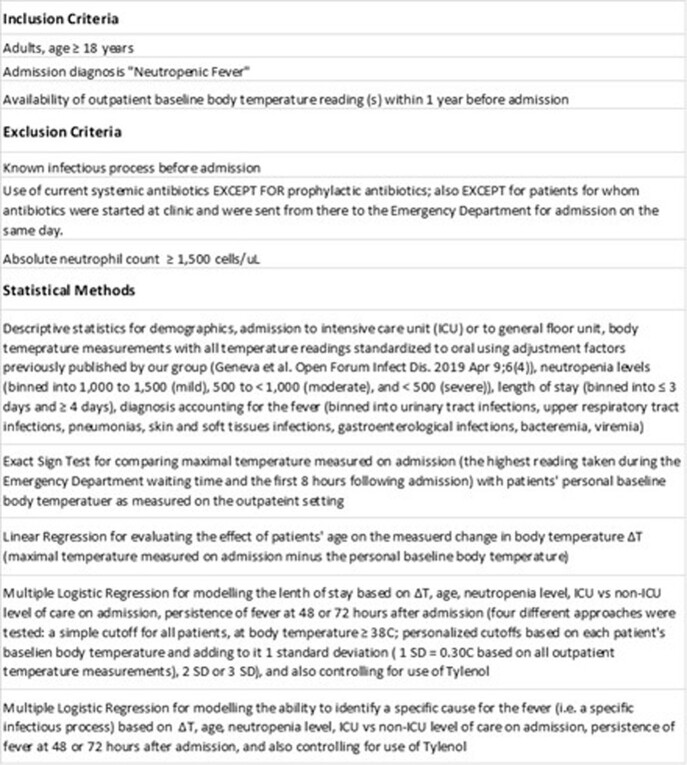
Inclusion and exclusion criteria and statistical methods.

**Results:**

Refer to Tables 2 and 3 for demographics and descriptive statistics of temperature readings, to Table 4 for advanced statistical analyses. Importantly, the average baseline body temperature was at 36.7C; the average fever on admission was at 38.1C; based on the ≥ 38C cutoff, only 24% of patients had persistent fever **over 48-72 hours** but based on personalized cutoffs at > 2 standard deviations (SDs) or > 3 SDs above their outpatient baseline, 54% and 34% had persistent fever, respectively; the etiology of fever was identified in 48% of patients, all of which constituted infections; our multiple regression model demonstrated that a longer length of stay (LOS) of ≥ 4 days was predicted by larger deviation from baseline body temperature at admission and independently by fever persistence, after correcting for age, neutropenia level, and need for ICU level of care on admission. A similar model could not predict the ability to identify a fever-explaining diagnosis.

**Figure:**
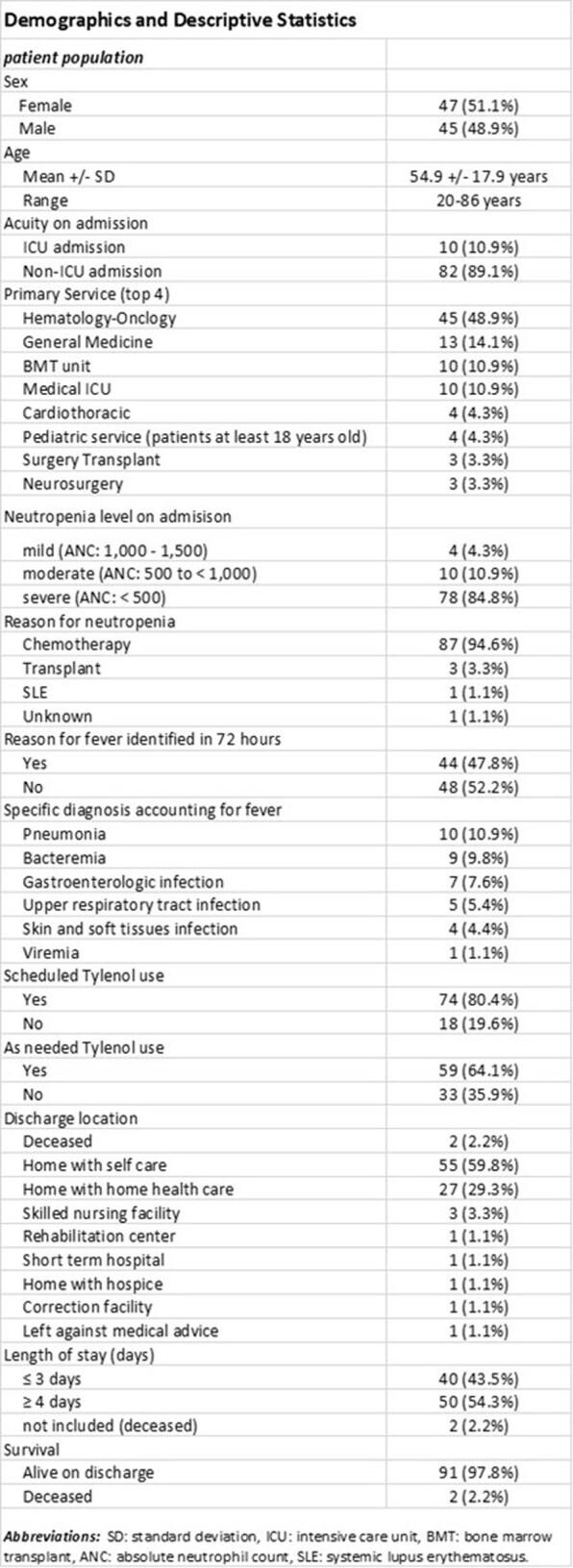

**Figure:**
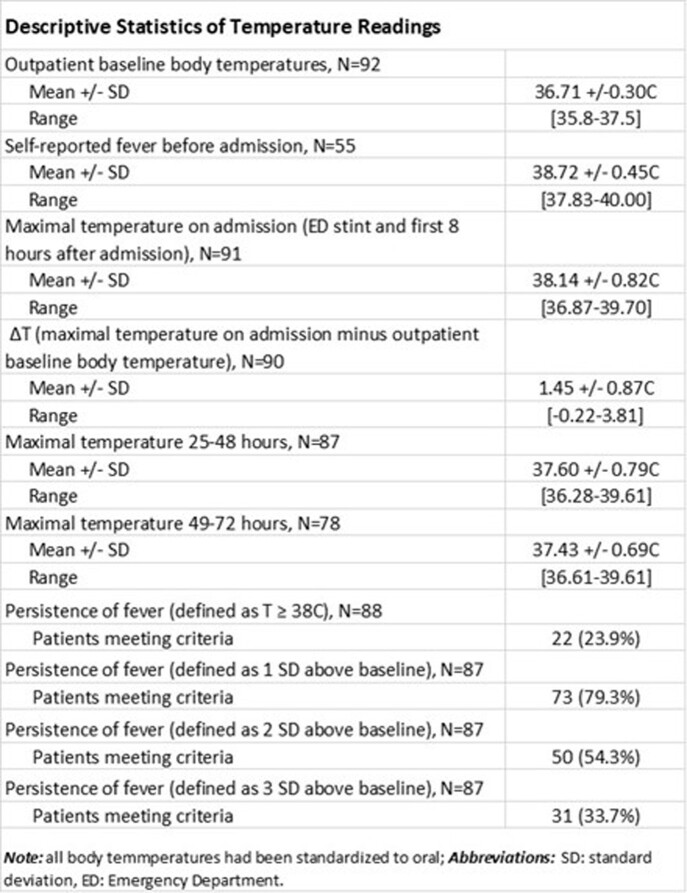

**Figure:**
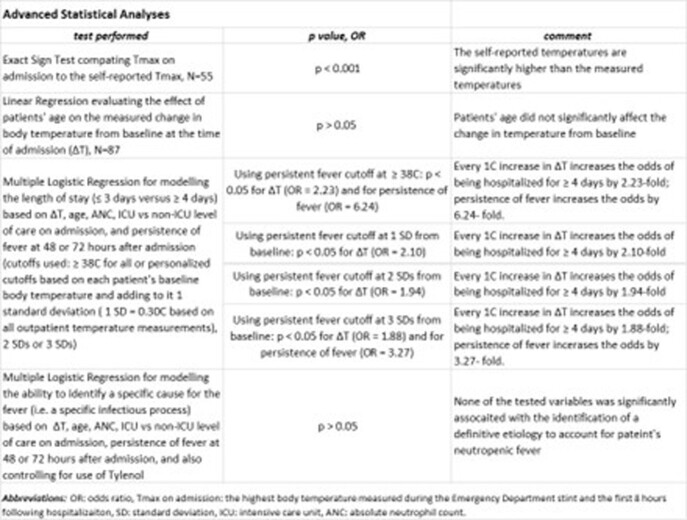

**Conclusion:**

Given the average outpatient baseline body temperature of 36.7 +/- 0.3C, at 2 standard deviations (SDs) above this baseline, only 3% of patients would be at the 38C cutoff for fever, at 3 SDs, it would be 20%, thus rendering the standard 38C cutoff too high to be useful in identifying many neutropenic fever cases and supporting the use of personalized cutoffs based on patient’s baseline temperature. Further, consideration of the specific deviation from patients’ baseline body temperature could serve as a predictor for hospital LOS in patients admitted with neutropenic fever.

**Disclosures:**

**All Authors**: No reported disclosures.

